# Supplementary material for: Removal of Transgenes and Evaluation of Yield Penalties in Genome Edited Bacterial Blight Resistant Rice Varieties
Source: Plant Biotechnol J. 2025 Oct 7;24(2):939–53. doi: 10.1111/pbi.70332 (PMC12906797; doi:10.1111/pbi.70332)
Supplement: Supplementary file 1 — Figure S1: pbi70332‐sup‐0001‐FigureS1.pdf. [file PBI-24-939-s001.pdf]

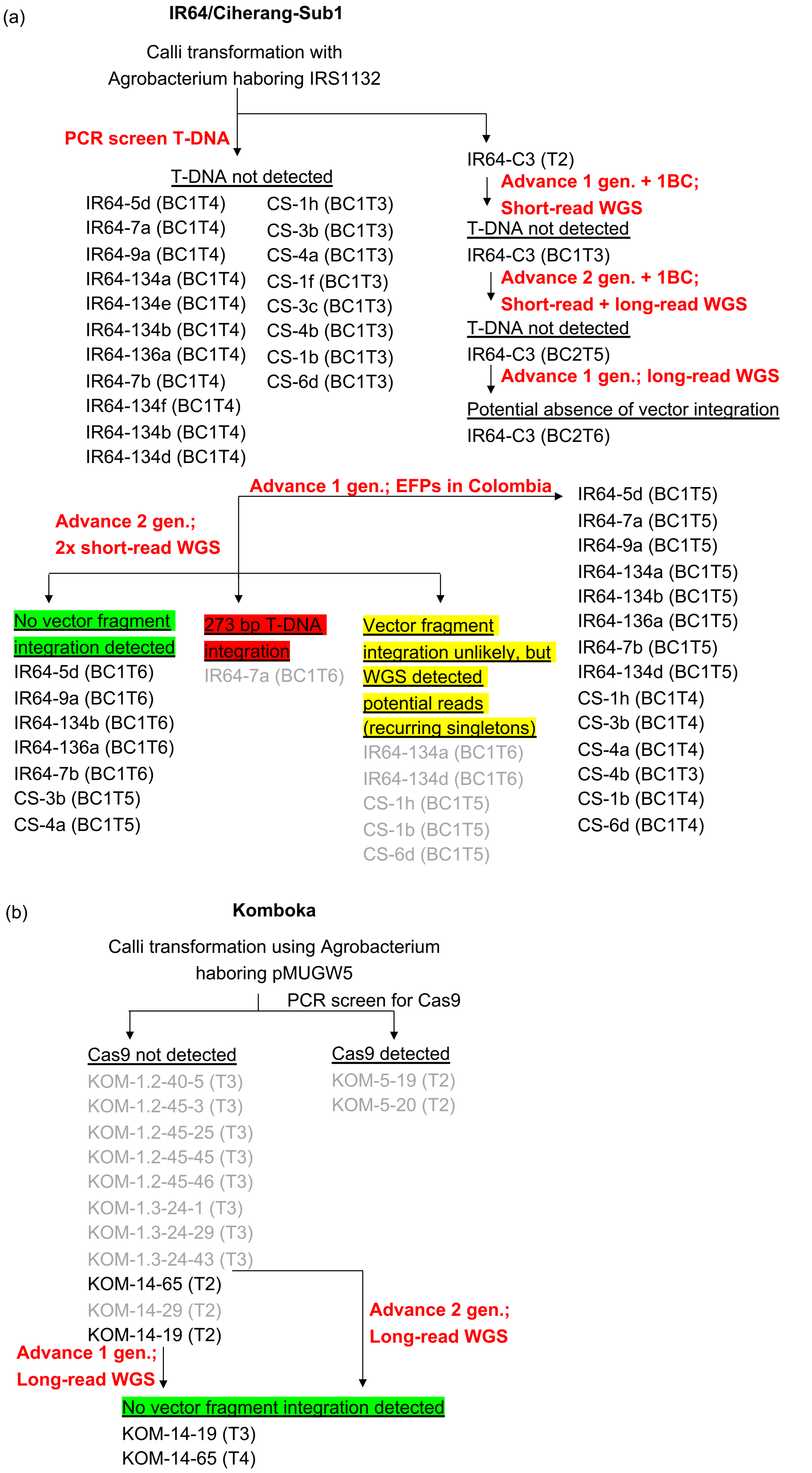

Figure S1: Overview of tests performed in different generations of GE'd A) IR64 and Ciherang-Sub1 lines; and B) Komboka lines
