# Supplementary material for: Removal of Transgenes and Evaluation of Yield Penalties in Genome Edited Bacterial Blight Resistant Rice Varieties
Source: Plant Biotechnol J. 2025 Oct 7;24(2):939–53. doi: 10.1111/pbi.70332 (PMC12906797; doi:10.1111/pbi.70332)
Supplement: Supplementary file 2 — Figure S2: pbi70332‐sup‐0002‐FigureS2.pdf. [file PBI-24-939-s008.pdf]

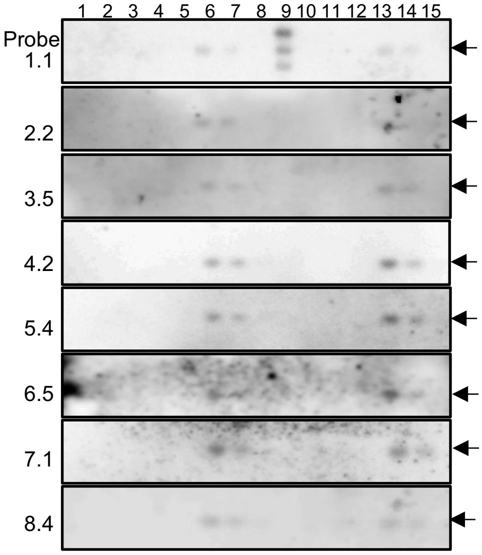

Lane	Line ID
1	IR64-9a
2	IR64-136a
3	IR64-C3
4	IR64-H12
5	IR64 WT
6	IR64 WT + 12 pg IRS1132
7	IR64 WT + 6 pg IRS1132
8	IR64 WT + 1.2 pg IRS1132

Lane	Line ID
9	DIG ladder
10	CS-3b
11	CS-4
12	CS WT
13	CS WT + 12 pg IRS1132
14	CS WT + 6 pg IRS1132
15	CS WT + 1.2 pg IRS1132

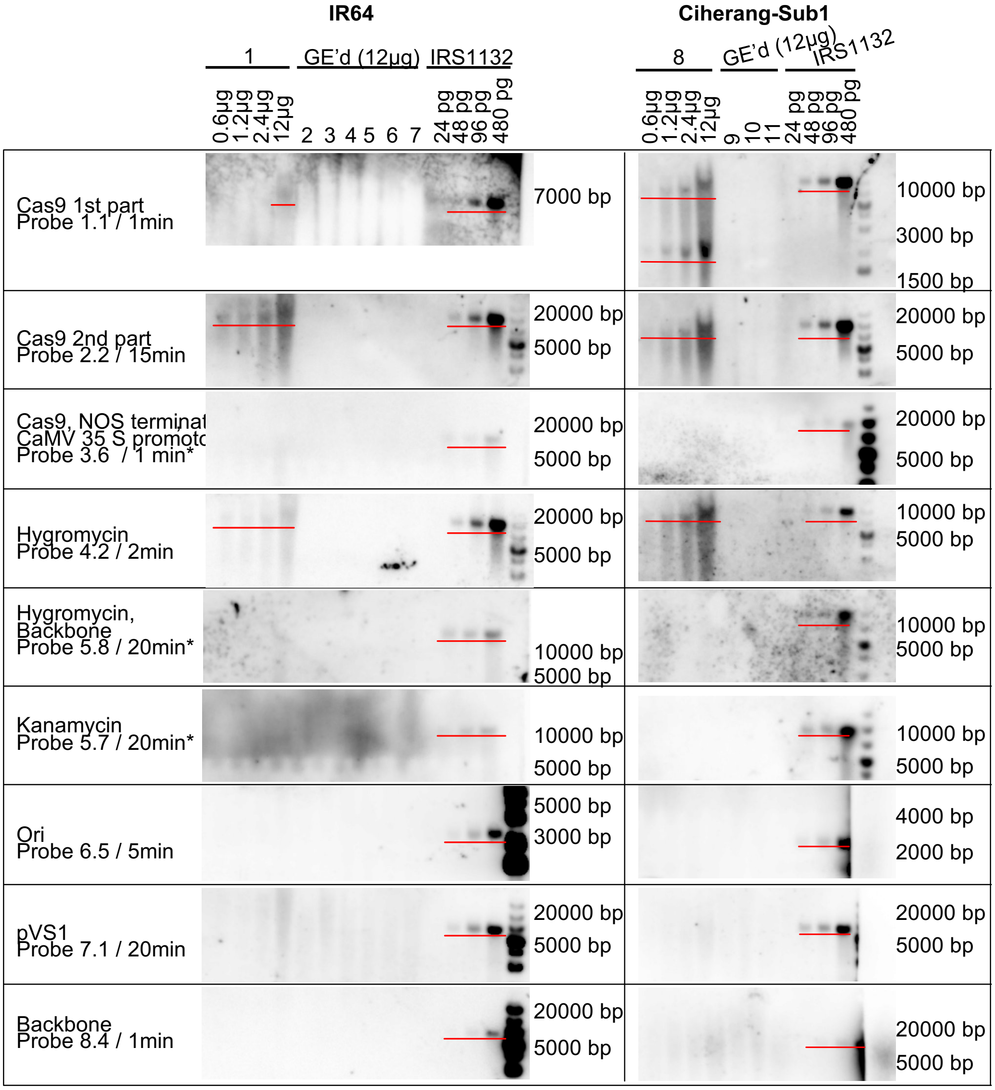

Lane	Line ID
1	IR64-136b
2	IR64-5d
3	IR64-7b
4	IR64-9a
5	IR64-136a
6	IR64-C3
7	IR64 WT
8	CS-3b
9	CS-3b
10	CS-4
11	CS WT

Figure S2: DNA gel blot with overlapping probe for the detection of transgene in GE'd lines
DNA gel blot of GE'd IR64 and Ciherang-Sub1 lines using the overlapping probes (Table S1). IR64-136b and CS-3b are GE'd parental lines with vector integration as positive controls for GE'd lines of respective cultivars. IRS1132 indicate the vector (positive) control. Arrows and red lines indicate fragment of expected size. DNA samples used for each lane in the DNA gel blots indicated in table on the right of the blots.
