# Supplementary figures and images for: Removal of Transgenes and Evaluation of Yield Penalties in Genome Edited Bacterial Blight Resistant Rice Varieties
Source: Plant Biotechnol J. 2025 Oct 7;24(2):939–53. doi: 10.1111/pbi.70332 (PMC12906797; doi:10.1111/pbi.70332)

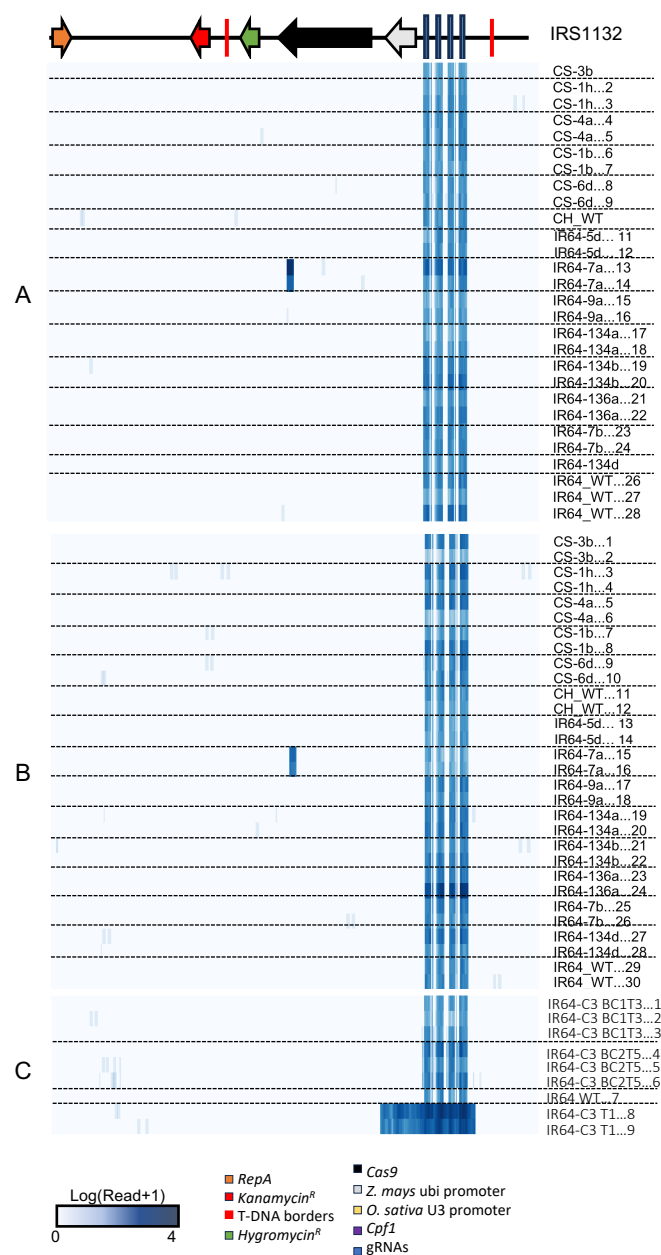

Supplement: Supplementary file 3 — Figure S3: pbi70332‐sup‐0003‐FigureS3.pdf. [file PBI-24-939-s006.pdf]

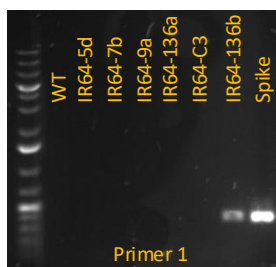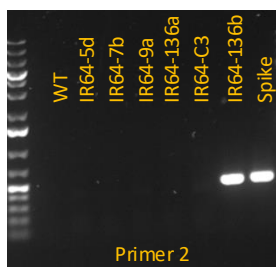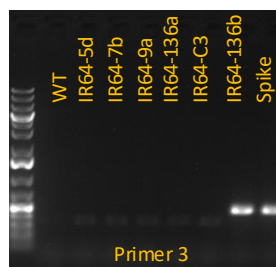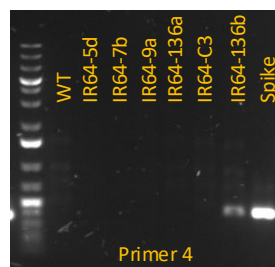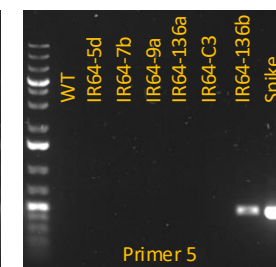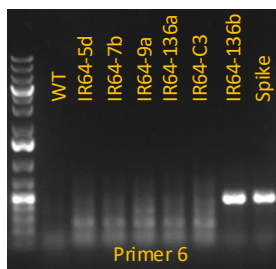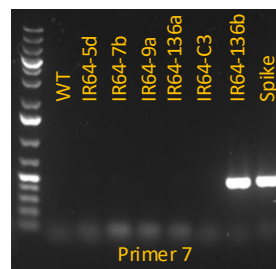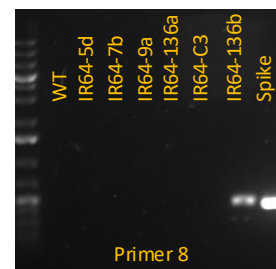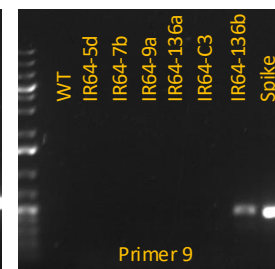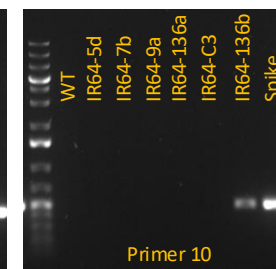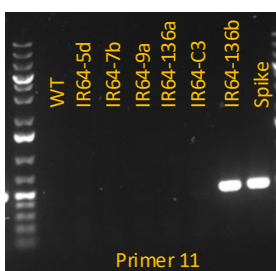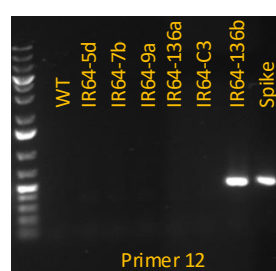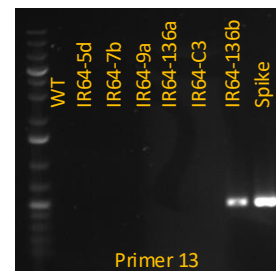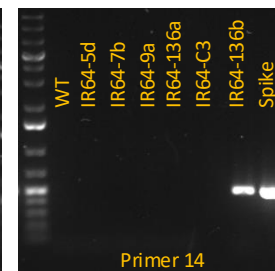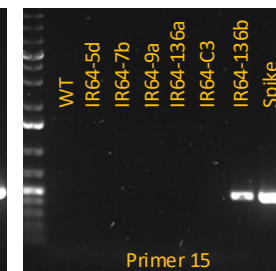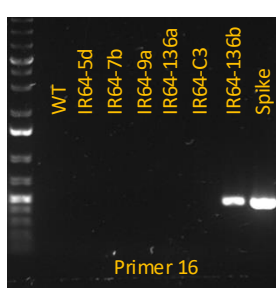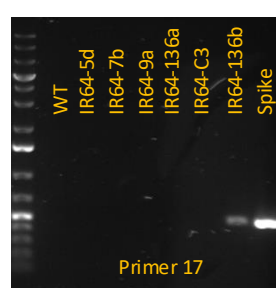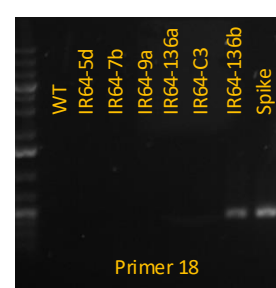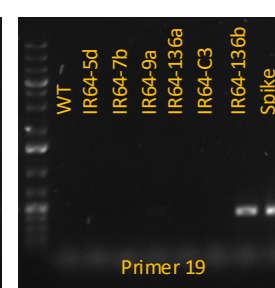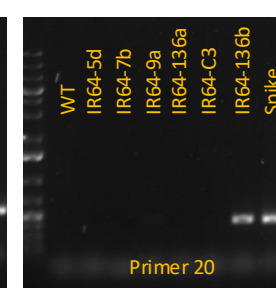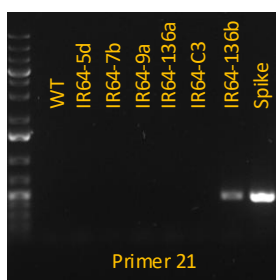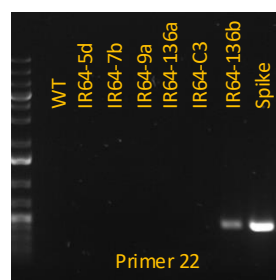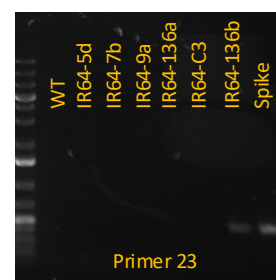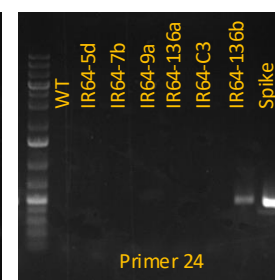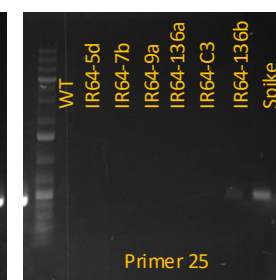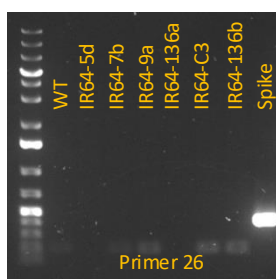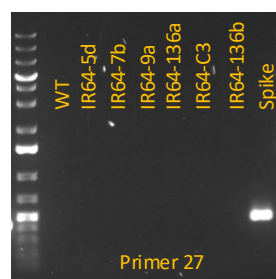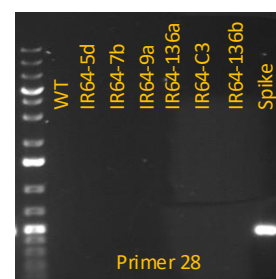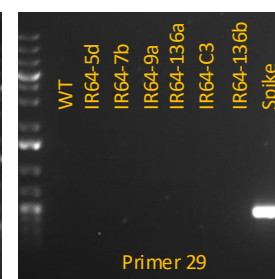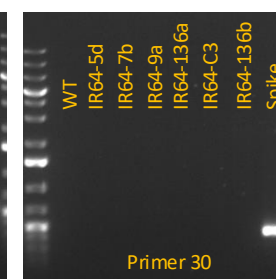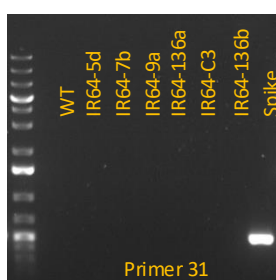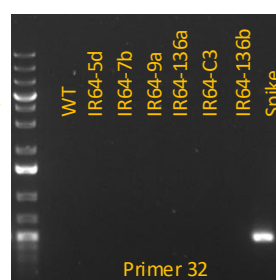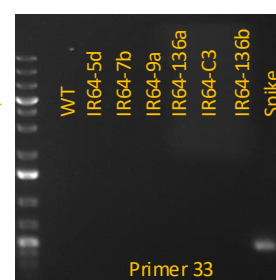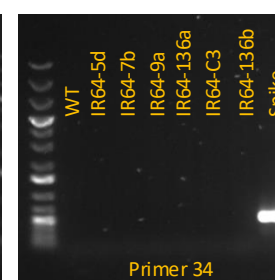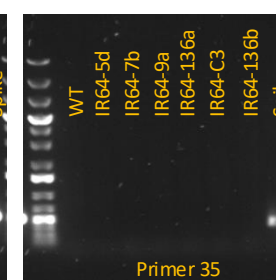

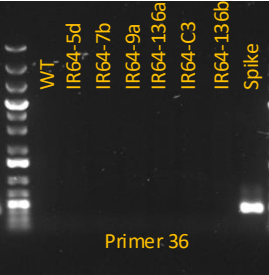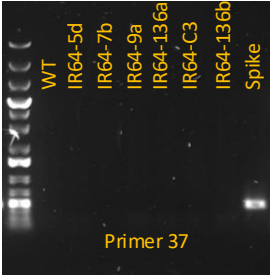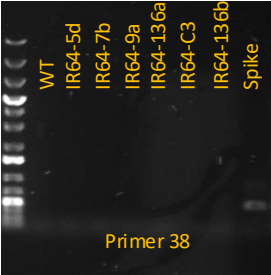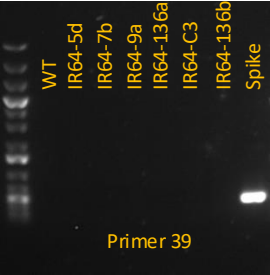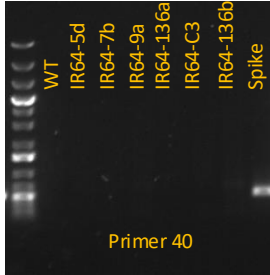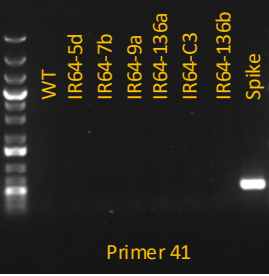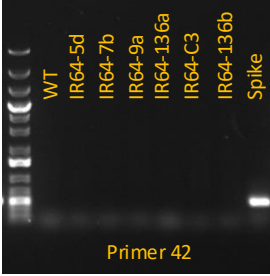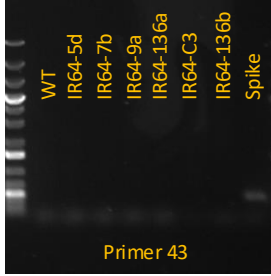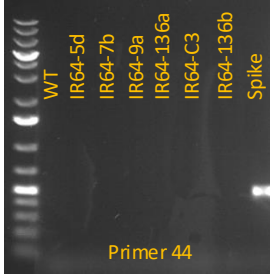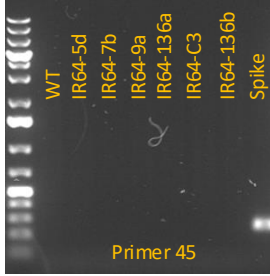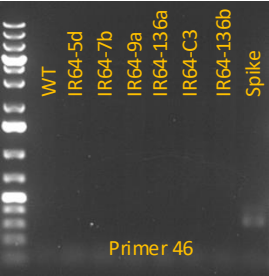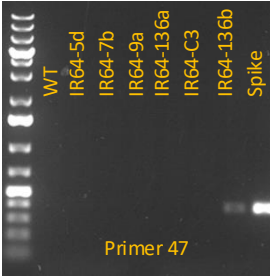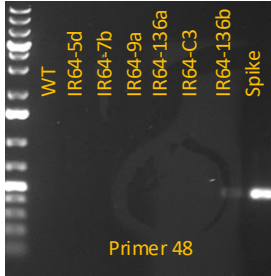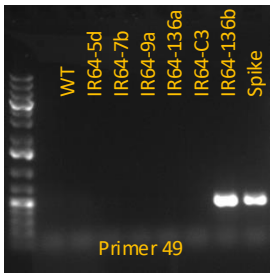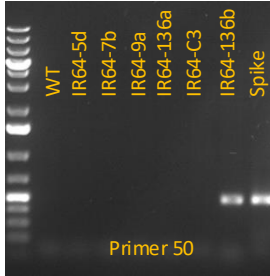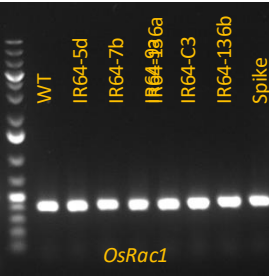

Supplement: Supplementary file 5 — Figure S5: pbi70332‐sup‐0005‐FigureS5.pdf. [file PBI-24-939-s004.zip › Fig S5C.pdf]

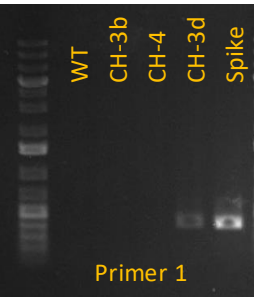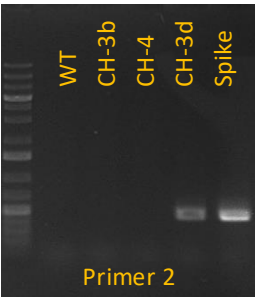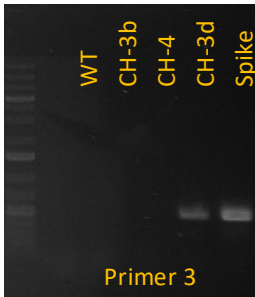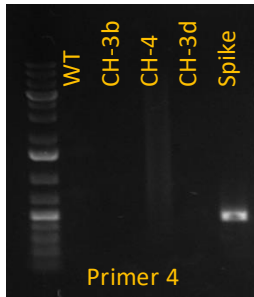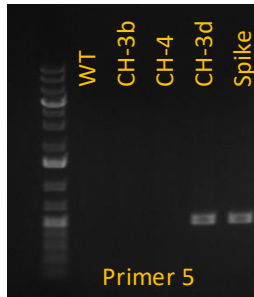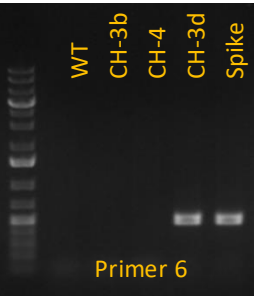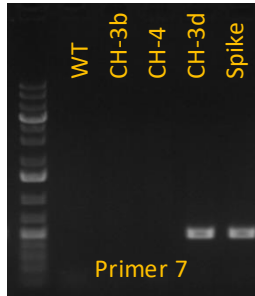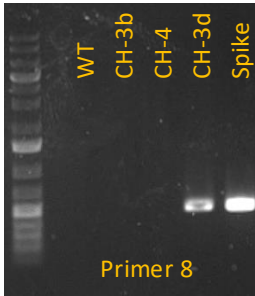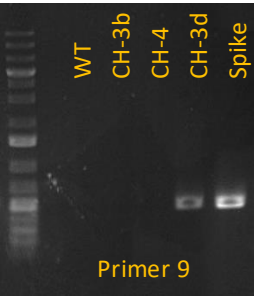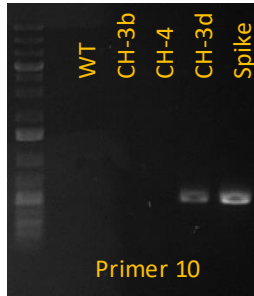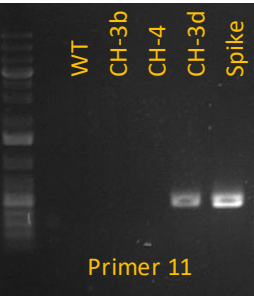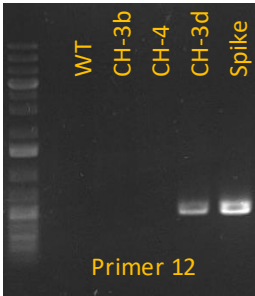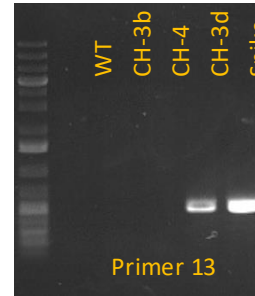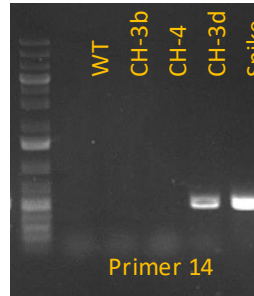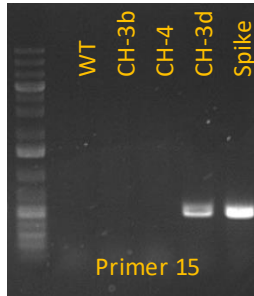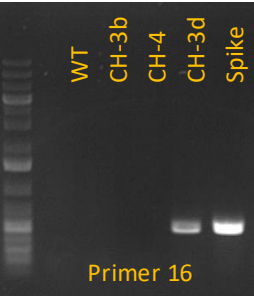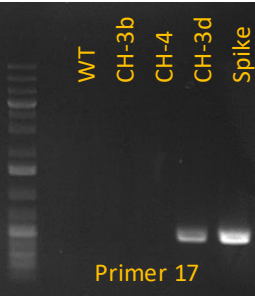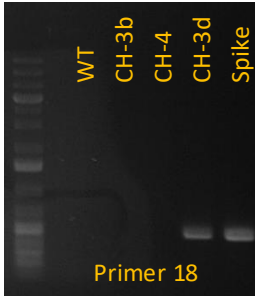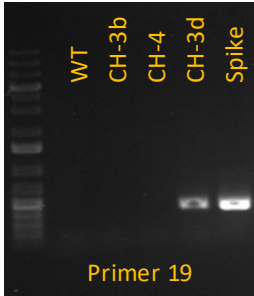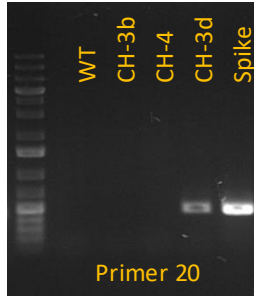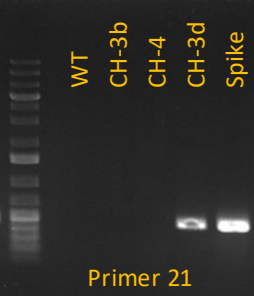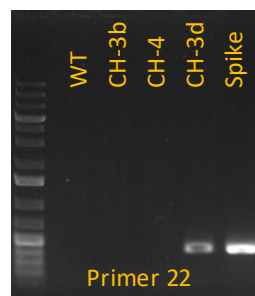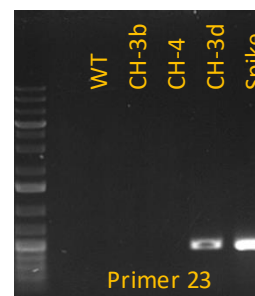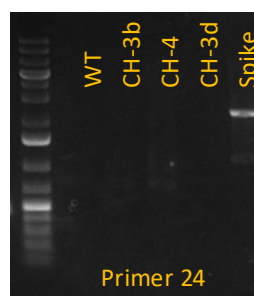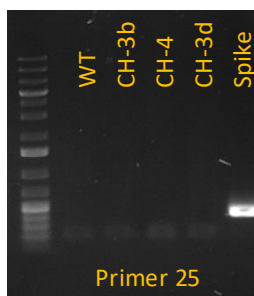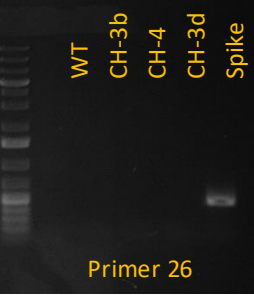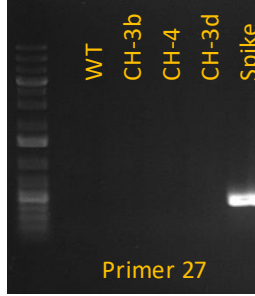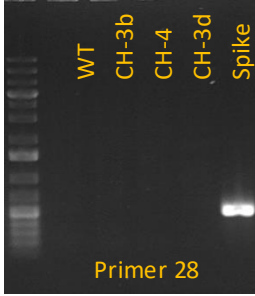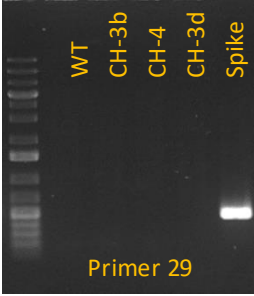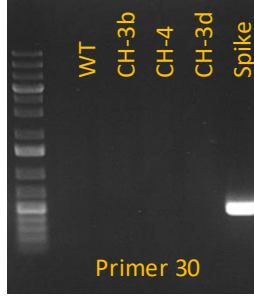

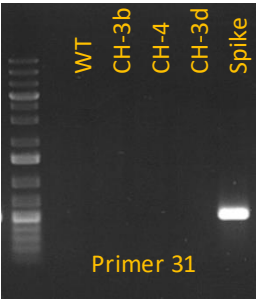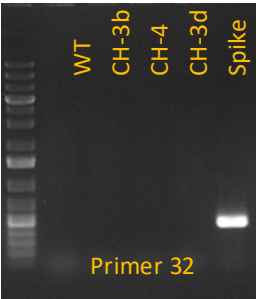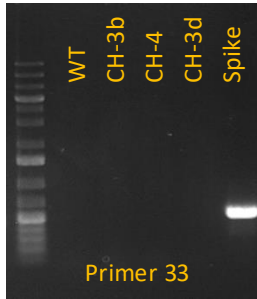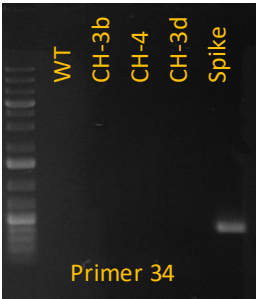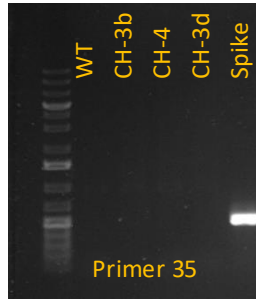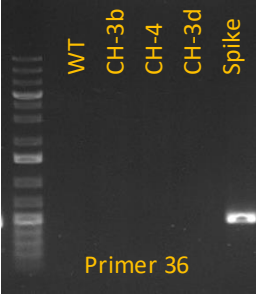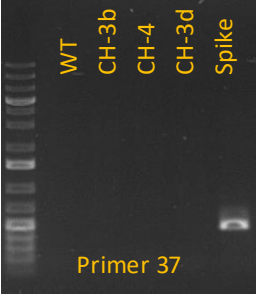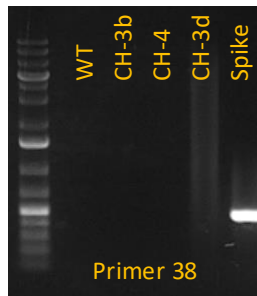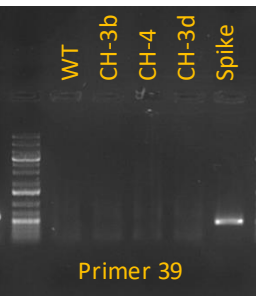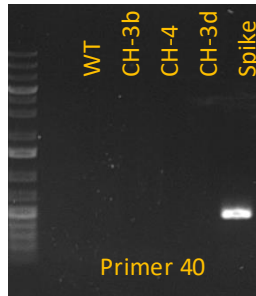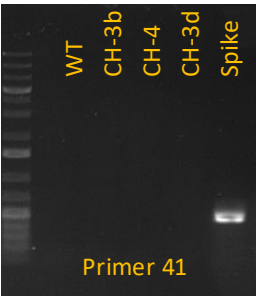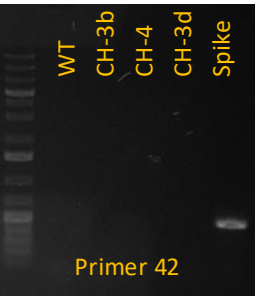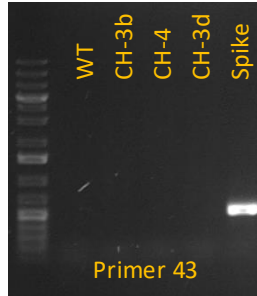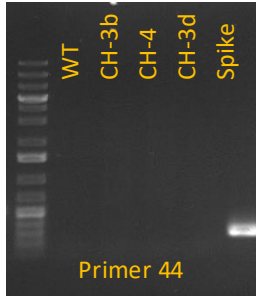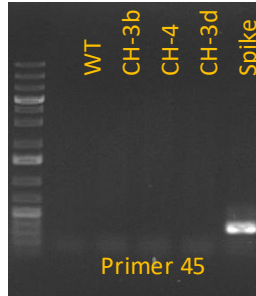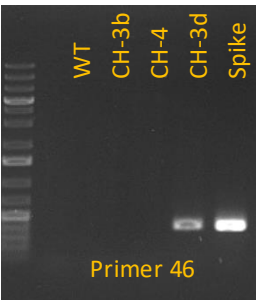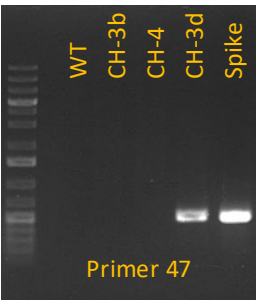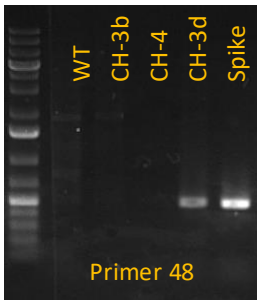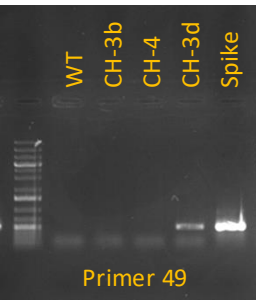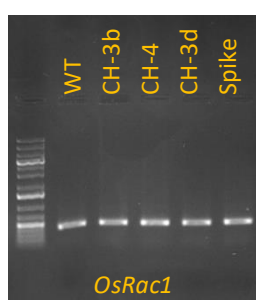

Supplement: Supplementary file 5 — Figure S5: pbi70332‐sup‐0005‐FigureS5.pdf. [file PBI-24-939-s004.zip › Fig S5D.pdf]

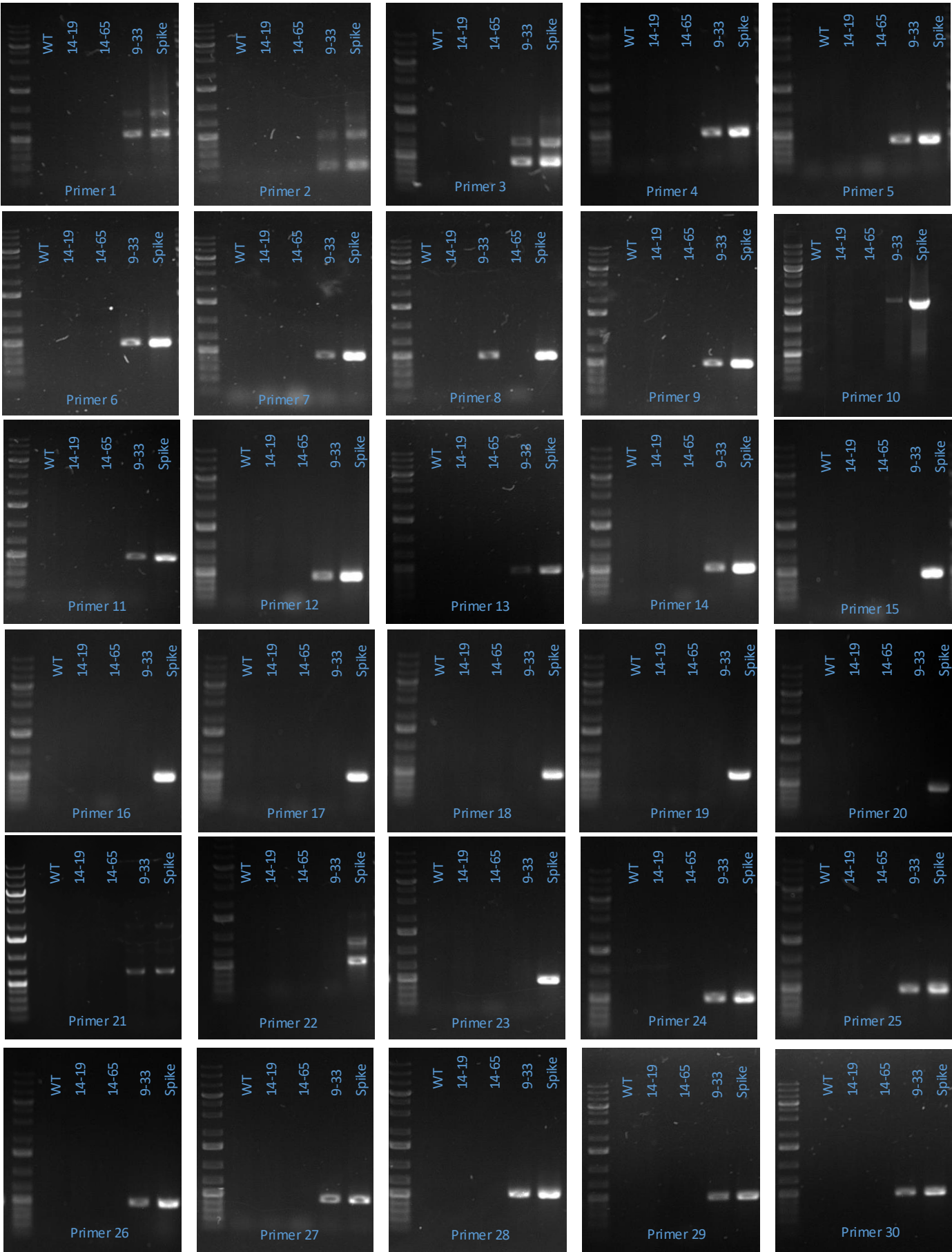

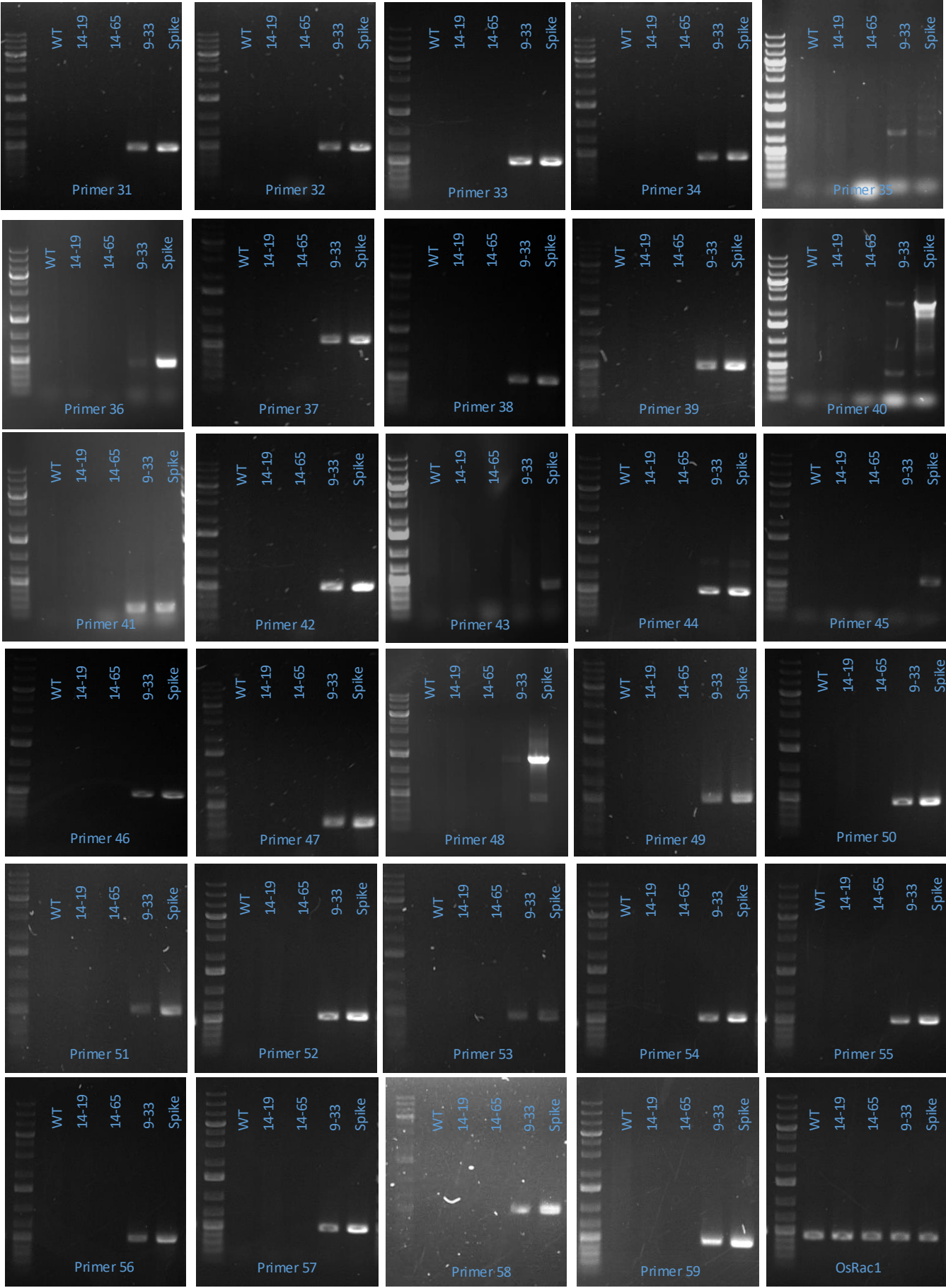

Supplement: Supplementary file 5 — Figure S5: pbi70332‐sup‐0005‐FigureS5.pdf. [file PBI-24-939-s004.zip › Fig S5E.pdf]
