# Supplementary material for: Removal of Transgenes and Evaluation of Yield Penalties in Genome Edited Bacterial Blight Resistant Rice Varieties
Source: Plant Biotechnol J. 2025 Oct 7;24(2):939–53. doi: 10.1111/pbi.70332 (PMC12906797; doi:10.1111/pbi.70332)
Supplement: Supplementary file 4 — Figure S4: pbi70332‐sup‐0004‐FigureS4.pdf. [file PBI-24-939-s007.pdf]

(a)

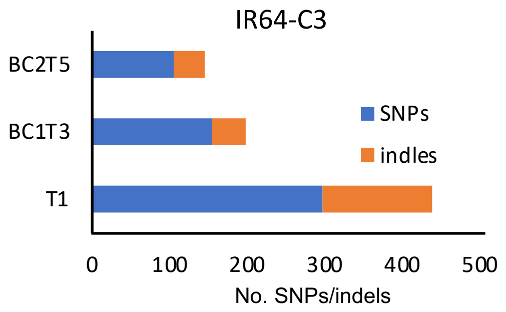

(b)

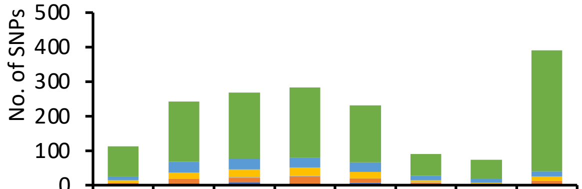

	IR64_C3	IR64-5a	IR64-9a	IR64-136a	IR64-7b	CS-3B	CS-4	KOM-14-19
Intergenic	86	174	191	203	164	63	56	350
Intron	12	32	33	28	28	13	11	15
Exon	7	18	22	25	19	6	3	13
Splice site	0	0	1	2	0	1	1	0
Missense	7	14	14	20	14	5	1	8
Silent	0	4	8	5	6	2	2	5

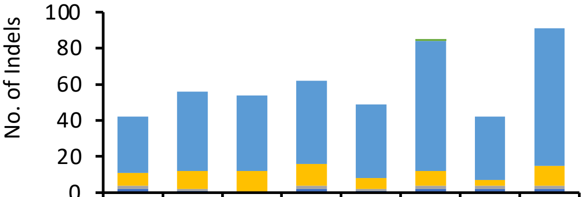

	IR64_C3	IR64-5a	IR64-9a	IR64-136a	IR64-7b	CS-3B	CS-4	KOM-14-19
Splice site	0	0	0	0	0	1	0	0
Intergenic	31	44	42	46	41	72	35	76
Intron	7	10	12	12	6	8	3	11
Exon	2	1	0	2	1	2	2	2
In Frame	0	0	0	0	0	0	0	0
Frameshift	2	1	0	2	1	2	2	2

(c)

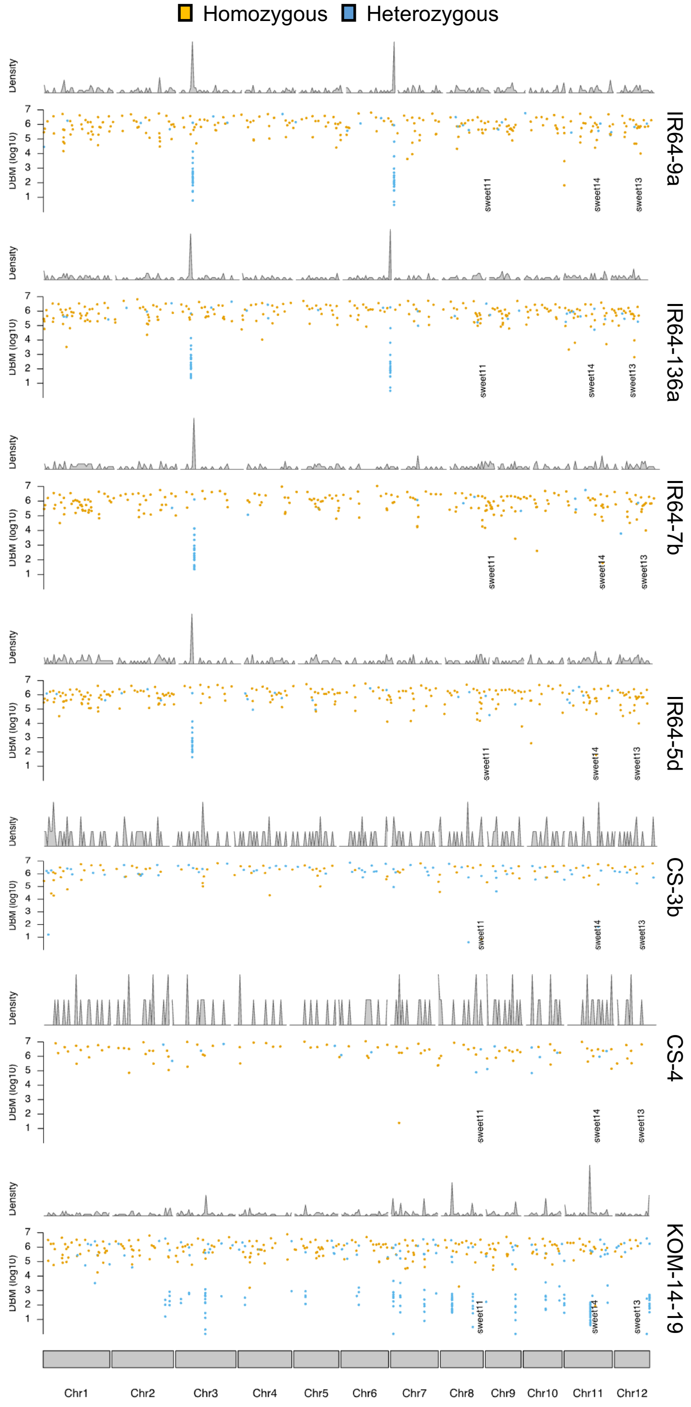

Figure S4: GE'd lines do not carry detrimental indels or SNPs

A) Number of SNPs and indels found in three generations of GE'd IR64-C3

B) SNPs and indels detected in the consensus genome sequence of indicated GE'd lines. Exact number of SNPs/indels indicated in the table below bar plots.

C) Density and distribution of SNPs and indels detected in each chromosome of GE'd line. Peaks above the dotplots indicate the density of SNPs/indels. Color of the dots indicate heterogenous SNPs (blue), or homogenous SNPs (yellow). Position of SNPs (chromosome) indicate on the X-axis.
