# Supplementary material for: Removal of Transgenes and Evaluation of Yield Penalties in Genome Edited Bacterial Blight Resistant Rice Varieties
Source: Plant Biotechnol J. 2025 Oct 7;24(2):939–53. doi: 10.1111/pbi.70332 (PMC12906797; doi:10.1111/pbi.70332)
Supplement: Supplementary file 5 — Figure S5: pbi70332‐sup‐0005‐FigureS5.pdf. [file PBI-24-939-s004.zip › Fig S5A-B.pdf]

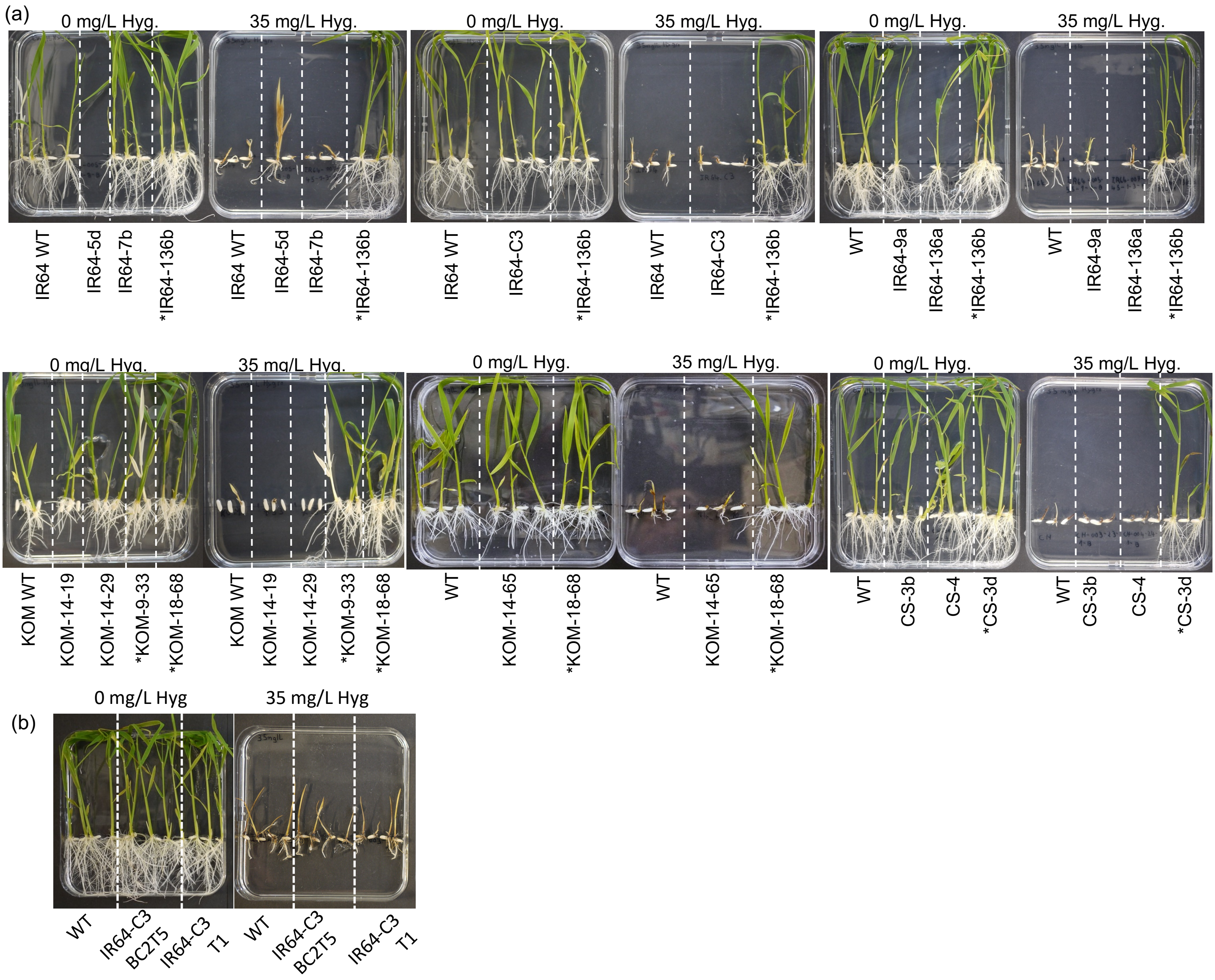

Figure S5: Absence of herbicide tolerance in GE'd lines.

A) Hygromycin test on GE'd, parental (positive control, indicated with *), and WT plants (negative control).

B) Hygromycin-tolerant gene silencing resulted in loss of hygromycin tolerance in transgene-positive GE'd IR64-C3 T1. WT: wild-type plant, IR64-C3 BC2T5: GE'd IR64 without vector intergration, IR64-C3 T1: GE'd IR64 with carrying T-DNA.

C) Gel electrophoresis images for overlap PCR on GE'd IR64 for the detection of vector IRS1132

D) Gel electrophoresis images for overlap PCR on GE'd Ciherang-Sub1 for the detection of vector IRS1132

E) Gel eletrophoresis images for overlap PCR on GE'd Komboka for the detection of vector pMUGW5
