# Supplementary material for: Removal of Transgenes and Evaluation of Yield Penalties in Genome Edited Bacterial Blight Resistant Rice Varieties
Source: Plant Biotechnol J. 2025 Oct 7;24(2):939–53. doi: 10.1111/pbi.70332 (PMC12906797; doi:10.1111/pbi.70332)
Supplement: Supplementary file 6 — Figure S6: pbi70332‐sup‐0006‐FigureS6.pdf. [file PBI-24-939-s002.pdf]

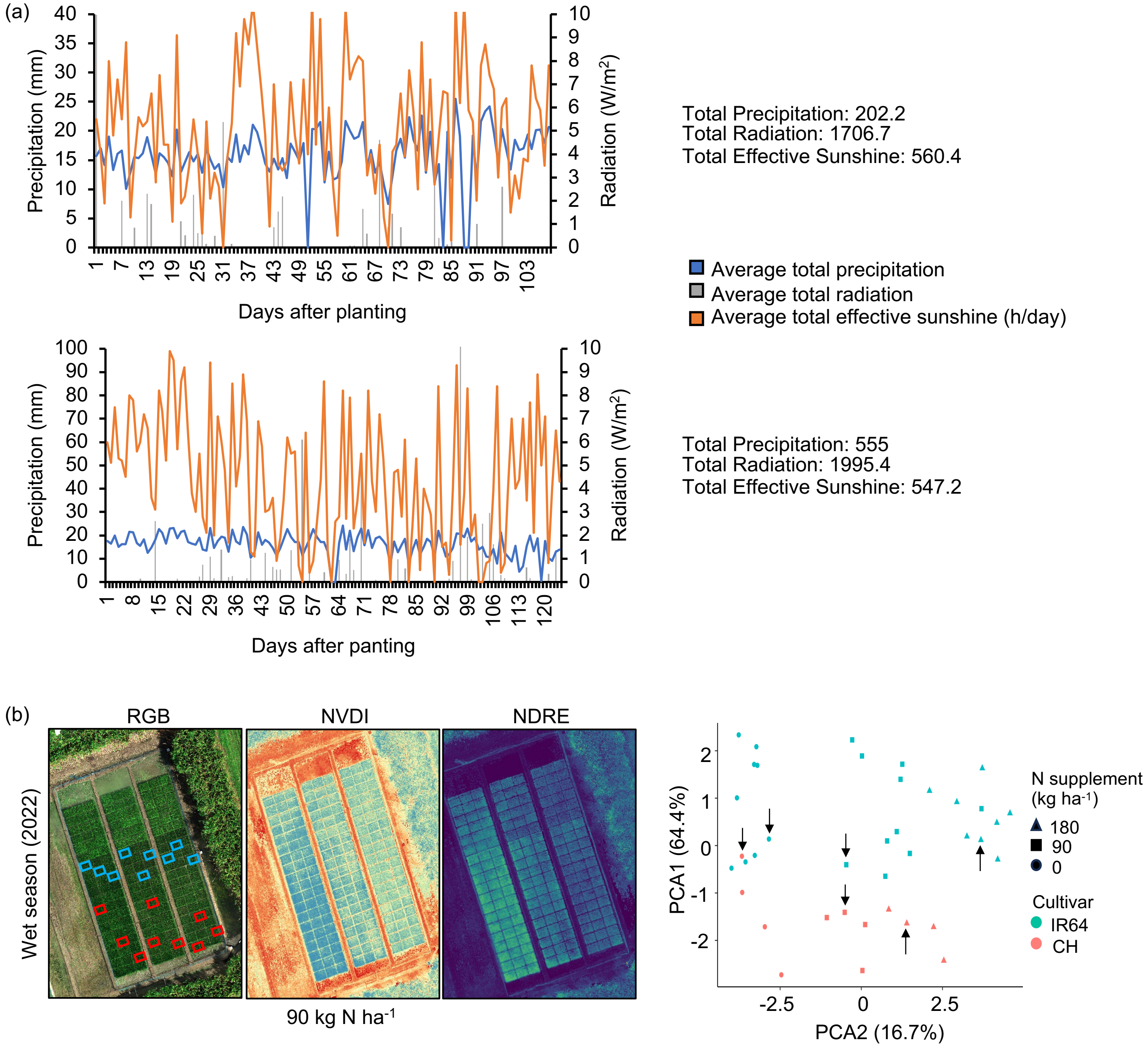

Figure S6: Metreological data during EFPs and agronomic performance of GE'd lines in EFPs.

A) Meteorological information from the CIAT, Colombia June 2020 dry season (top) and January 2021 rainy season (bottom) of EFPs.

B) RGB and multispectral images of the EFPs conducted in Colombia in the wet season of 2021 and its corresponding PCA analysis of EBE-edited and WT IR64 and Ciherang-Sub1 lines under 0%, 50%, and 100% nitrogen treatment. Arrows indicate datapoint for WT plants. Boxed in red indicate WT IR64 plants, boxed in blue indicates WT Ciherang-Sub1 plants.
