# Supplementary material for: Removal of Transgenes and Evaluation of Yield Penalties in Genome Edited Bacterial Blight Resistant Rice Varieties
Source: Plant Biotechnol J. 2025 Oct 7;24(2):939–53. doi: 10.1111/pbi.70332 (PMC12906797; doi:10.1111/pbi.70332)
Supplement: Supplementary file 7 — Figure S7: pbi70332‐sup‐0007‐FigureS7.pdf. [file PBI-24-939-s009.pdf]

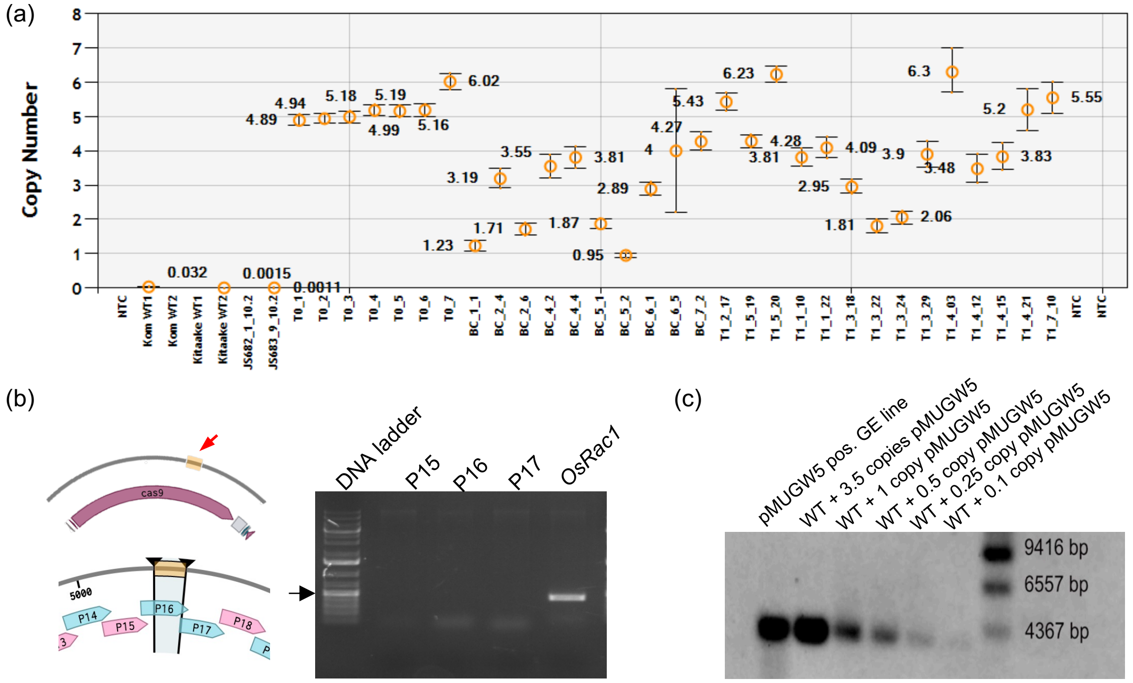

Figure S7: Evaluation of the efficiency of transgene analysis

A) Determination of Cas9 copy number in T0, T1, and BC1T1 GE'd Komboka via ddPCR. NTC- No template control, Komboka (Kom) and Kitaake WT as negative controls.

B) Overlap PCR failed to detect the presence of Cas9 fragment integrated into the plant's genome. Red arrow and region highlighted in orange indicates the 289 bp fragment from Cas9 incorporated into the genome of IR64-7a. Blue and pink arrows indicate the amplicon produced by indicated primer pairs. Gel electrophoresis image of PCR amplification of IR64-7a using P15, P16, and P17 primers (see scheme on left). *OsRac1* was used as a positive control. Black arrow indicates DNA ladder position for 500 bp.

C) Cpf1-positive transgenic Komboka and WT Komboka spiked with dilutions of pMUGW5 vector blot against DIG-labeled Cpf1 probe.
