# Supplementary material for: Removal of Transgenes and Evaluation of Yield Penalties in Genome Edited Bacterial Blight Resistant Rice Varieties
Source: Plant Biotechnol J. 2025 Oct 7;24(2):939–53. doi: 10.1111/pbi.70332 (PMC12906797; doi:10.1111/pbi.70332)
Supplement: Supplementary file 8 — Figure S8: pbi70332‐sup‐0008‐FigureS8.pdf. [file PBI-24-939-s003.pdf]

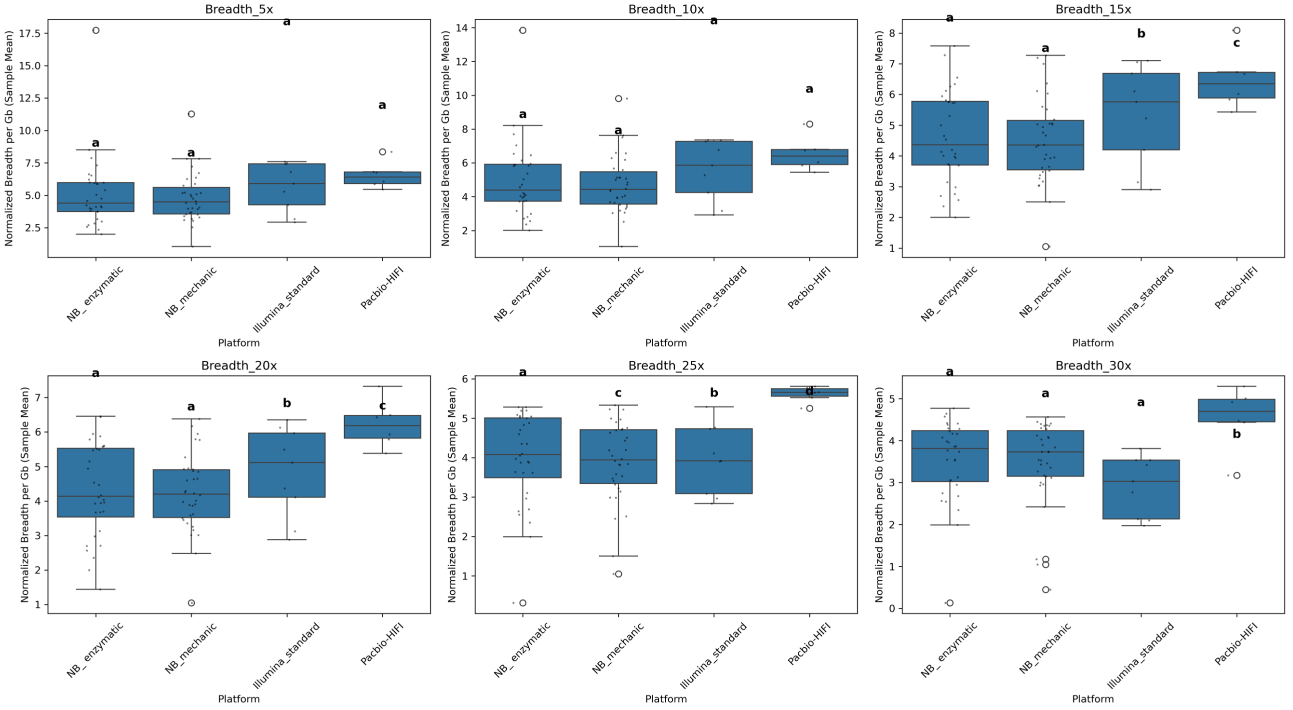

Figure S8: Evaluation of the coverage depth and breadth for the various protocols used for WGS of GE'd lines. An analysis of genome coverage uniformity for each WGS library. The fraction of bases meeting or exceeding certain depth thresholds (5x, 10x, 15x, 20x, 25x, and 30x) indicate the coverage breadth.
